# Actin-related protein Arp4 regulates euchromatic gene expression and development through H2A.Z deposition in blood-stage *Plasmodium falciparum*

**DOI:** 10.1186/s13071-020-04139-6

**Published:** 2020-06-17

**Authors:** Hui Liu, Xin-Yu Cui, Dan-Dan Xu, Fei Wang, Lin-Wen Meng, Yue-Meng Zhao, Meng Liu, Shi-Jun Shen, Xiao-Hui He, Qiang Fang, Zhi-Yong Tao, Ci-Zong Jiang, Qing-Feng Zhang, Liang Gu, Hui Xia

**Affiliations:** 1 Department of Microbiology and Parasitology, Bengbu Medical College, Bengbu, 233030 China; 2 The School of Life Sciences and Technology, Tongji University, Shanghai, 200092 China; 3 Research Center for Translational Medicine, Key Laboratory of Arrhythmias of the Ministry of Education of China, East Hospital, Tongji University School of Medicine, Shanghai, 200120 China; 4 Anhui Provincial Key Laboratory of Infection and Immunology, Bengbu Medical College, Bengbu, 233000 China; 5 Tsingtao Advanced Research Institute, Tongji University, Qingdao, 266071 China

**Keywords:** Malaria, *Plasmodium falciparum*, Arp4, Chromatin structure, Gene regulation

## Abstract

**Background:**

Malaria caused by *Plasmodium* spp. is still a major threat to public health globally. The various approaches to developing new antimalarial agents rely on the understanding of the complex regulatory mechanisms of dynamic gene expression in the life-cycle of these malaria parasites. The nuclear members of the evolutionarily conserved actin-related protein nuclear (ARP) superfamily are the major components of nucleosome remodelling complexes. In the human malaria parasite *Plasmodium falciparum*, bioinformatics analysis has predicted three ARP orthologues: PfArp1, PfArp4 and PfArp6. However, little is known about the biological functions of putative PfArp4. In this study, we aimed to investigate the function and the underlying mechanisms of *PfArp4* gene regulation.

**Methods:**

A conditional gene knockdown approach was adopted by incorporating the glucosamine-inducible glmS ribozyme sequence into the 3’ UTR of the *PfArp4* and *PfArp6* genes. The transgenic parasites PfArp4-Ty1-Ribo, PfArp6-Ty1-Ribo and pL6-PfArp4-Ty1::PfArp6-HA were generated by the CRISPR-Cas9 technique. The knockdown effect in the transgenic parasite was measured by growth curve assay and western blot (WB) analysis. The direct interaction between PfArp4 and PfArp6 was validated by co-IFA and co-IP assays. The euchromatic gene expression mediated through H2A.Z (histone H2A variant) deposition and H3K9ac modification at promoters and regulated by *PfArp4*, was determined by RNA-seq and ChIP-seq.

**Results:**

The inducible knockdown of *PfArp4* inhibited blood-stage development of *P. falciparum*. *PfArp4* and *PfArp6* were colocalized in the nucleus of *P. falciparum* parasites. *PfArp4* gene knockdown altered the global transcriptome. PfArp4 protein colocalized with the histone variant H2A.Z and euchromatic marker H3K9ac in intergenic regions. The inducible downregulation of *PfArp4* resulted in the depletion of H2A.Z and lower H3K9ac levels at the upstream regions of eukaryotic genes, thereby repressing the transcriptional abundance of H2A.Z-dependent genes.

**Conclusions:**

Our findings suggest that *PfArp4* regulates the cell cycle by controlling H2A.Z deposition and affecting centromere function, contributing to the understanding the complex epigenetic regulation of gene expression and the development of *P. falciparum.*
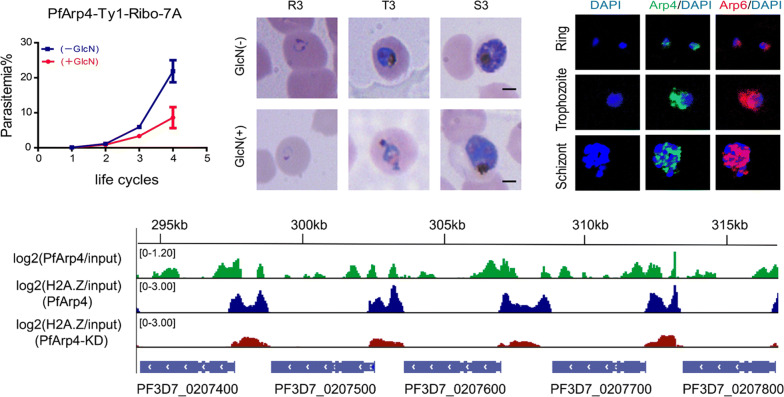

## Background

Malaria caused by *Plasmodium* parasites is still a major threat to public health globally. In 2018, there were approximately 229 million malaria cases worldwide, which resulted in 435,000 deaths [1]. The emerging and rapidly spreading drug resistance to artemisinin derivatives has led to globally diminishing malaria control [2, 3]. The various approaches to developing new antimalarial tools rely on the understanding of the complex regulatory mechanisms of dynamic gene expression in the life-cycle of malaria parasites. Epigenetic regulation of gene expression is a basic strategy utilized by most eukaryotic cells during physiological processes of development and proliferation. The epigenetic mechanisms involve DNA methylation, mediation of local chromatin structure by histone tail modification, noncoding RNAs (ncRNAs), nuclear architecture and newly discovered RNA epigenomes created by modifications such as m^6^A [4] or m5C [5]. In addition, nucleosome remodelling by selective deposition and dynamic exchange of some histone variants, such as H2A.Z at the untranslated regions is a general way to regulate gene expression in eukaryotes.

In the human malaria parasite *Plasmodium falciparum*, the local chromatin structure marked by different histone modifications is the major factor determining the activity of gene transcription during infection, development and pathogenesis in the host. For virulence-associated antigenic variant genes such as *var*, *rifin* and *stevor* [6], the heterochromatic islands in the host genome enriched by trimethylation of lysine 9 in histone H3 (H3K9me3) and coupled with heterochromatin protein 1 (HP1) control the transcriptional silencing of most variant genes, whereas the singular active member is modified by acetylation of lysine 9 (H3K9ac) or trimethylation of lysine 4 (H3K4me3) on histone H3 at the promoter region. This arrangement of chromatin structures in the nucleus establishes mutually exclusive expression of various virulence genes. In addition, previous experiments have identified four different histone variants (H2A.Z, H2Bv, H3.3 and CenH3, which is a centromeric histone variant) in *P. falciparum* [7, 8]. Genome-wide immunoprecipitation coupled with high-throughput sequencing (ChIP-seq) analysis revealed that H2A.Z demarcates the euchromatic intergenic regions that are dynamically marked by H3K9ac and H3K4me3. Further profiling of histone modification and mRNA abundance levels showed that only H2A.Z and H3K9ac correlated with gene transcriptional activity over the course of the intraerythrocytic cycle, suggesting that these two histone markers represent euchromatic genes. In particular, the exchange of H2A.Z at the upstream promoter region of *var* genes is associated with the singular expression and switching in the next generation as a genetic memory.

In *P. falciparum*, some chromatin-associated factors have been found to interact with different chromatin markers [9]. For instance, PfHP1 recruited by H3K9me3 modification contributes to the formation and maintenance of heterochromatin islands, thereby controlling the expression of heterochromatic genes, including virulence genes, invasion genes, and the master regulator of sexual commitment, *ap2-g*. In addition, for the euchromatic genes, PfBDP1 (*P. falciparum* bromodomain protein1) and AP2-I have been shown to coregulate the transcription of invasion genes *via* specific interactions between the bromodomain of PfBDP1 and the acetylated lysine at promoter regions. However, to date, little is known about the regulatory factors that determine the selective deposition and dynamic exchange of H2A.Z at promoter regions of euchromatic genes. In other eukaryotes, Arp6 is an acritical component of the SRCAP (Snf2-related CREBBP activator protein) chromatin remodelling complex, which deposits H2A.Z onto chromatin. In budding yeast, H2A.Z/Htz1 is located at the nucleosome in the transcription start site [10, 11] and prevents the diffusion of heterochromatin proteins [12, 13]. Experiments have shown that Arp6 contributes to transcriptional regulation by exchanging H2A for Htz1 [14]. Moreover, actin not only plays a part in the filamentous cytoskeleton [15] but also in various chromatin remodelling pathways in the nucleus. However, it remains unclear whether actin-related proteins are involved in the regulatory functions of H2A.Z during gene expression in *P. falciparum*.

Actin-related proteins (ARPs) in eukaryotes are a relatively conserved family of proteins [16, 17]. This family is divided into multiple subfamilies, and most eukaryotic cells contain at least eight actin-related proteins [18]. In general, Arp1-3 is present in the cytoplasm, whereas Arp4 to Arp9 have been detected in the nucleus, often as part of a multi-subunit chromatin-modifying enzyme complex, i.e. chromatin remodelling and histone acetyltransferase (HAT) complexes, or in DNA replication and other chromatin transactions [19–21]. Budding yeast Arp4 was the first identified nuclear ARP and was found to be a component of the NuA4 (nucleosome acetyltransferase of H4) complex [22] and chromatin remodelling complexes Ino80 [23] and SWR1 [24]. In particular, Arp4 and Arp8 bind to core histones, suggesting that they may promote the interaction between their respective complexes and nucleosomes [25]. For *P. falciparum*, a comparative genome analysis predicted only three ARP: orthologues PfArp1, PfArp4 and PfArp6 [26]. Here, we aimed to investigate the putative function of the two nuclear ARPs (PfArp4 and PfArp6) in gene regulation and the underlying mechanisms.

## Methods

### Plasmid construction

A pLN-ENR-GFP plasmid was modified by the replacement of *gfp* with the 1.5-kb C-terminus of *PfArp4* fused with the Ty1-Ribo sequence by in-fusion cloning. The resulting vector, *pL6-PfArp4-Ty1-Ribo*, was used to transfect *P. falciparum* 3D7 parasites. For the *pL6-PfArp6-Ty1-Ribo* construct, the 1.5-kb C-terminus of *PfArp4* was replaced with the 1.5-kb C-terminus of *PfArp6*. For the *pL6-PfArp6-Ty1-HA* construct, the Ty1-Ribo fragment of the *pL6-PfArp6-Ty1-Ribo* construct was replaced with a sequence encoding the HAx3-Ty1x3 tag *via Asc I*/*Afl II*. The *pL6-PfArp4-Ty1::PfArp6-HA* plasmid was modified by replacing *PfArp4* with the 600 bp C-terminus of *PfArp4* fused with the Ty1x3 tag and replacing *PfArp6* with the 600 bp C-terminus of *PfArp6* fused with the HAx3 tag by in-fusion cloning.

### Parasite culture and transfection

The *P. falciparum* 3D7 strain was cultivated and synchronized as described previously [27]. Two hundred microlitres of ring-stage parasites at a parasitaemia level of 5% were transfected with 100 µg of plasmid DNA by electroporation as described previously [28]. When the parasitaemia levels reached 8–10%, WR99210 (Sigma-Aldrich, St Louis, MO, USA) and BSD (Sigma-Aldrich) (blasticidin-S deaminase drugs) were added to the culture for the selection of transgenic parasites. Approximately 21 days after the transfection, the transgenic parasites appeared in the culture. The genomic DNA of the transgenic parasites was extracted for verification of gene fusion events by PCR.

### Phylogenetic analysis

Sequences were aligned using ClustalX v1.83, and alignments were manually adjusted using the BioEdit alignment editor (https://www.bioedit.com). Distance trees were made using both neighbour-joining and Poisson methods with a MEGA 5.05 software package (https://www.megasoftware.net). A maximum-likelihood (ML) tree of the Arp4 domains and Arp6 domains was constructed using the MEGA package (https://www.megasoftware.net/). Bootstrapping was performed for 1000 replicates. The accession numbers of Arp4 and Arp6 in are listed in Additional file 1: Table S1.

### Western blot analysis

Synchronized parasites at a parasitaemia level of 2–3% were collected and lysed with 0.15% saponin. After washing twice with 1% PBS, the cells were resuspended in 1× SDS loading buffer (Bio-Rad, Hercules, CA, USA). Since the molecular weight of PfArp4 and PfArp6 is approximately 62 kDa and 121 kDa, respectively, an 8% SDS-PAGE gel was used to isolate the proteins. The antibodies used in the experiment were rabbit anti-HA (1:2000; Abcam, Cambridge, UK), mouse anti-Ty1 (1:1000; Sigma-Aldrich), and rabbit anti-PfAldolase (1:1000; Abcam, Cambridge, UK). An ECL western blot kit (GE Healthcare, Atlanta, GA, USA) was used to develop the blots.

### Immunofluorescence assays (IFA)

Immunofluorescence assays were performed as previously described [29]. Synchronized *P. falciparum* parasites at a parasitaemia level of 2% were collected. The erythrocytes were washed once with incomplete 1640 medium at room temperature, and then, 0.15% saponin was added and mixed well for 10–15 min on ice to lyse the erythrocytes. The purified parasites were fixed with 4% paraformaldehyde (Sigma-Aldrich). After washing with 1× PBS, the cells were resuspended for immunofluorescence analysis. The antibody was diluted with 0.1% BSA. The mouse anti-Ty1 antibody was diluted to 1:500, the rabbit anti-HA antibody was diluted to 1:500, and the Alexa Fluor 488 combined anti-rabbit antibody was diluted to 1:500.

### Co-immunoprecipitation (co-IP)

Co-IP was performed as described previously [29]. Briefly, asynchronized parasite cultures with parasitaemia levels of 2–3% were collected and lysed with 0.15% saponin. The released parasites were washed with 1× PBS and resuspended in three volumes of lysis buffer (2 mM EDTA; 0.5 mM PMSF; 25 mM Tris-Cl, pH 7.5; 100 mM KCl; 0.05% NP-40; and 1× protease inhibitor cocktail (Thermo Fisher Scientific, Waltham, MA, USA)) and subjected to sonication at the highest power for 4 min (30 s on, 30 s off) intervals with a sonicator (Diagenode SA, Liège, Belgium). The separated supernatant was immediately incubated overnight with magnetic beads that had been previously conjugated with the HA antibody (Abcam) at 4 °C. The beads were then washed twice with IPP500 (500 mM NaCl, 0.05% NP-40, and 10 mM Tris-Cl, pH 8.0) and once with 1× PBS. Then, the bound proteins were eluted with sample buffer for the SDS-PAGE and western blot analysis. Western blot analysis using anti-PfAldolase, anti-HA and anti-Ty1 antibodies verified the enrichment of the targeted proteins.

### RNA-seq

RNA isolation, mRNA enrichment and library construction were performed as described using 15 cycles of library amplification [30]. Briefly, synchronized parasites were harvested for extraction of total RNA with a Direct-zol RNA kit (Zymo Research, California, USA). mRNA was enriched by poly(A) selection with KAPA mRNA capture beads (KAPA Biosystems, Boston, USA) and fragmented to approximately 300–400 nucleotides (nt) in length. Then, all subsequent steps were performed in accordance with a KAPA stranded mRNA-seq kit (KAPA Biosystems) on an Illumina platform and sequenced on an Illumina HiSeq X Ten system.

### RNA-seq data analysis

Low-quality and adaptor sequences were trimmed from the reads using Cutadapt (v1.16) [31] with the following parameters: -a AGATCGGAAGAGC-A AGATCGGAAGAGC, –trim-n -m 50, and -q 20,20. RNA sequencing reads were mapped to the *P. falciparum* 3D7 genome (Pf 3D7 v32, obtained from PlasmoDB) using HISAT2 strand-specific mode (v2.1.0) [32] with the following parameters: –rna-strandness RF, –dta, –no-discordant, –no-mixed, and –no-unal. Then, mapped reads were subsequently assembled into transcripts guided by the PlasmoDB gff annotation files (Pf 3D7 v32) using feature counts (v1.6.1) [33] with the following parameters: -M -p -B -C for all; -s2 for sense transcripts; -s1 for antisense transcripts. Sense read counts were merged for library normalization between conditions. FPKM (fragment per kilobase of transcript per million reads mapped) of sense transcripts was calculated using R (v3.5.1) (https://www.r-project.org). Genes with a minimum of twofold changes (PfArp4 KD *versus* G7) were considered differentially expressed genes in the two samples.

### Gene ontology analysis

Gene ontology (GO) enrichment was performed using R (v3.5.1). The GO term database was downloaded from PlasmoDB (https://plasmodb.org/plasmo/). GO terms with a *P*-value ≥ 0.05 (Fisher’s exact test) and an enriched gene number ≥ 5 were considered to be enriched. We next classified our GO terms into different functional categories for better understanding.

### Chromatin immunoprecipitation and high-throughput sequencing (ChIP-seq)

ChIP-seq analysis was performed as described previously [34]. Synchronized ring-stage (~ 20 hpi) parasites of PfArp4-Ty1-Ribo were used for a ChIP assay. The antibody against Ty1 antibody (Sigma-Aldrich) was used in this study. Briefly, the nuclei were isolated by incubation with 2 ml of cold lysis buffer for 30 min on ice, followed by Dounce homogenization for 200 strokes. The centrifuged (14,000× rpm, 10 min) nuclei were resuspended in 150 μl of SDS lysis buffer for sonication. Chromatin was sheared into 200–500 bp fragments with a Bioruptor (Diagenode SA). A total of 250 μl of chromatin supernatant was incubated overnight with 1 μg of Ty1 antibody at 4 °C, and mouse IgG (Sigma-Aldrich) was used as a control. The eluted DNA was purified by phenol/chloroform/isoamyl alcohol extraction for ChIP-seq. To prepare the sequencing libraries, purified DNA was end repaired, extended with 3′ A overhangs and ligated to barcoded NextFlex adapters (Bio Scientific, Austin, TX, USA). Libraries were amplified using KAPA HiFi Hot Start ready mix (KAPA Biosystems). Amplified libraries were size-selected for 300–600 bp using 2% agarose gels. The final libraries were sequenced on an Illumina HiSeq X Ten system to generate 150-bp paired-end reads.

### ChIP-seq data analysis (histone modifications (HM) and Arp4)

Low-quality and adaptor sequences were trimmed from the reads using Cutadapt (v1.16) [31] with the following parameters: -a AGATCGGAAGAGC-A AGATCGGAAGAGC, –trim-n -m 50, and -q 20,20. Then, the reads were mapped to the *P. falciparum* 3D7 genome (Pf 3D7 v32, obtained from PlasmoDB) using Bowtie2 (v2.3.4.3) [35] with following parameters: -N 0, –no-discordant,–no-mixed, –no-unal. SAMtools software (v1.9) [36] was used to transfer the mapping results from the SAM format to the position-sorted BAM format. Next, the duplicated reads were removed by markedup in sambamba (v0.6.8) [37]. Then, the BAM files were converted to BigWig files using bamCoverage from the deepTools suite (v3.1.3) [38] with the following parameters: –normalize Using RPKM and –binSize 25. The BigWig files from the ChIP sample were normalized to the input sample by BigWigCompare from the deepTools suite (v3.1.3) with the following parameters: –operation log2 and –pseudocount 1. The Integrative Genomics Viewer (IGV) [39] was used to show the signals of the histone modification marks in certain genomic regions in track view. The histone modification and Arp4 peaks were called with MACS2 [40] using the following parameters: -g 2.33e7 and -f BAMPE.

### The distribution of HMs and Arp4 signals around the gene body and centromere

The distribution of HMs and Arp4 signals around the gene body and centromere were generated by compute Matrix and plot Profile in the deep Tools suite (v3.1.3) [38]. To generate these data, all gene bodies were scaled to the same length of 1000 bp. A total of 2000 bp upstream of ATG and 2000 bp downstream of the stop codon were also included, and all centromeres were scaled to the same length of 1000 bp with 1000 bp upstream of ATG and 1000 bp downstream of the stop codon. The input data files for compute Matrix and plot Profile were the BigWig files of the ChIP signals normalized to the input sample (see the above “ChIP-seq data analysis”).

## Results

### Inducible knockdown of *PfArp4* inhibited the blood-stage development of *P. falciparum*

**Fig. 1:**
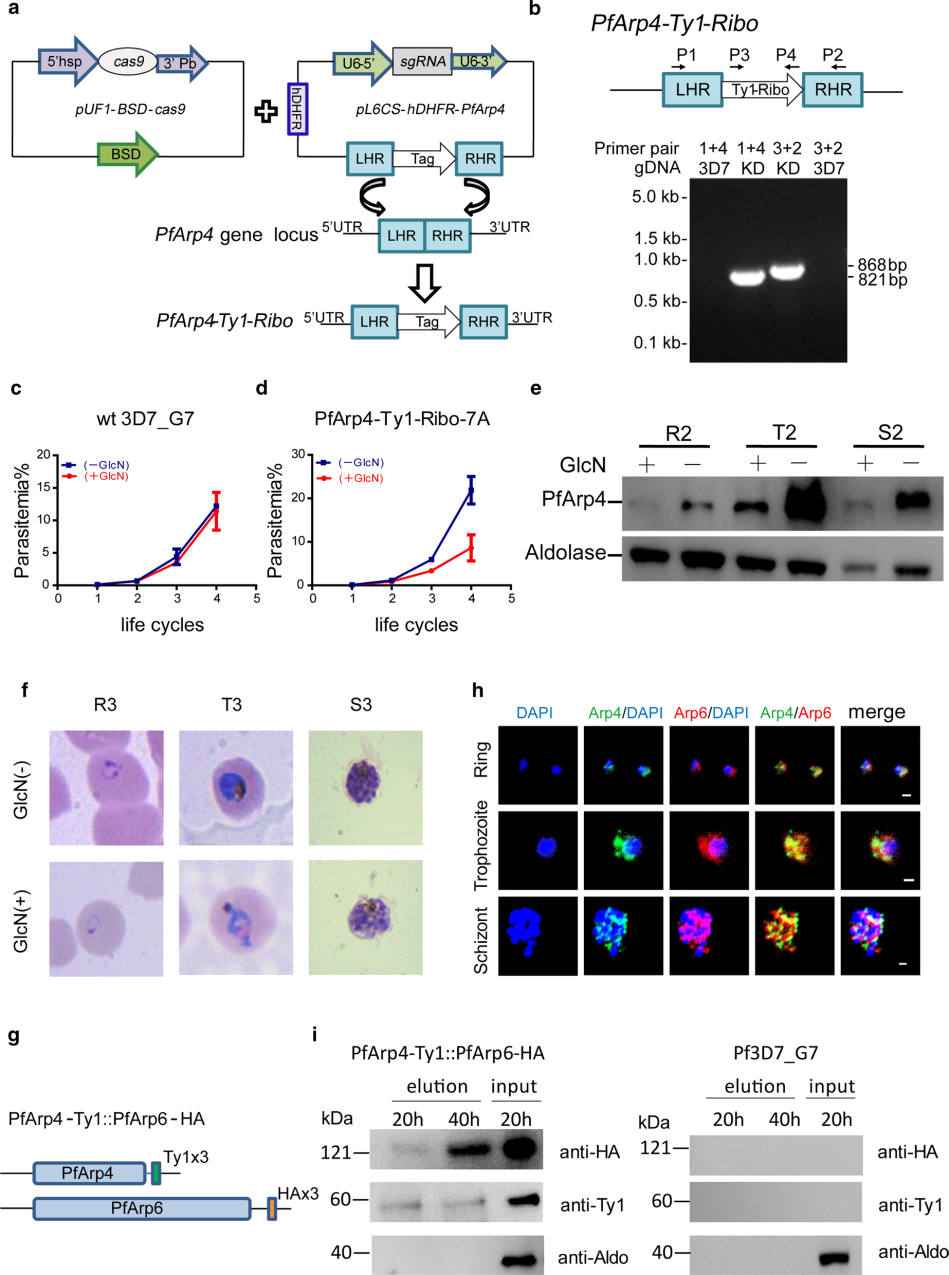
The nuclear PfArp4 is an essential protein in *P. falciparum.***a** Schematic representation of generation of transgenic PfArp4-Ty1-Ribo line. Co-transfection of the plasmid *pUF1-BSD-cas9* with *pL6CS-hDHFR-PfArp4* leads to integration of the *PfArp4-Ty1-Ribo* containing cassette in the endogenous locus. **b** PCR validation of PfArp4-Ty1-Ribo lines. The sequences of primer pairs are listed in Additional file 10: Table S4. **c** Growth curve assay of wild-type 3D7-G7 parasites with or without GlcN in the culture (*n* = 3, bars are SD). **d** Growth curve assay of PfArp4-Ty1-Ribo (7A clone) parasites with or without GlcN in the culture (*n* = 3, bars are SD). **e** Western blot of protein extracts from ring, trophozoite and schizont stage PfArp4-Ty1-Ribo parasites with (+) or without (−) GlcN in the culture for 2 cycles. **f** Representative Giemsa-stained blood smears of ring (R, 8–12 h), trophozoite (T, 28–32 h) and schizont (S, 40–44 h) stagePfArp4-Ty1-Ribo lines with (+) or without (−) GlcN in the culture for 3 cycles. **g** Schematic representation of the dual-tagging transgenic parasite line, PfArp4-Ty1::PfArp6-HA. **h** Co-IFA analysis of PfArp4-Ty1::PfArp6-HA line at ring, trophozoite, or schizont, respectively. **i** Co-IP analysis of PfArp4-Ty1::PfArp6-HA line and 3D7_G7 line (negative control) with anti-PfAldolase, anti-HA and anti-Ty1 antibodies for IP. *Abbreviations*: BSD, blasticidin-S deaminase; sgRNA, a synthetic single-guide RNA; hDHFR, human dihydrofolate reductase; RHL, 3′ flanking fragment for crossover recombination; LHL, 5′ flanking fragment for crossover recombination; Tag, insertion sequence. *Scale-bars*: **f**, 2 μm; **h**, 1 μm

In the genome of the *P. falciparum* 3D7 strain, two genes were predicted to encode the highly evolutionarily conserved PfArp4 (PF3D7_1422800) and PfArp6 (PF3D7_0719300) proteins. We constructed phylogenetic trees to trace the evolutionary relationship of PfArp4 and PfArp6 with their orthologues in other eukaryotes. The two Arp proteins in the genus *Plasmodium* formed a unique clade among eukaryotic organisms (Additional file 2: Figure S1, Additional file 3: Figure S2, Additional file 4: Figure S3). To study the potential roles of PfArp4 and PfArp6 in parasite biology, we first aimed to knockout the *PfArp4* and *PfArp6* genes by a frame shift strategy with the CRISPR-Cas9 technique [41], but it failed after at least three independent transfections using two different sgRNAs. This outcome suggests that the two genes are essential for parasite survival. Hence, the conditional gene knockdown approach was adopted by incorporating the glucosamine inducible *glmS* ribozyme sequence into the 3′ untranslated region of the *PfArp4* and *PfArp6* genes (Fig. 1a, Additional file 5: Figure S4a). Using this genetic manipulation system, we expected to downregulate the mRNA abundance at the post-transcriptional level upon the addition of glucosamine (GlcN) in the culture. After transfection, drug selection and limiting dilution cloning, we successfully obtained transgenic parasite clones of PfArp4-Ty1-Ribo and PfArp6-Ty1-Ribo (Fig. 1b, Additional file 5: Figure S4b).

**Fig. 2:**
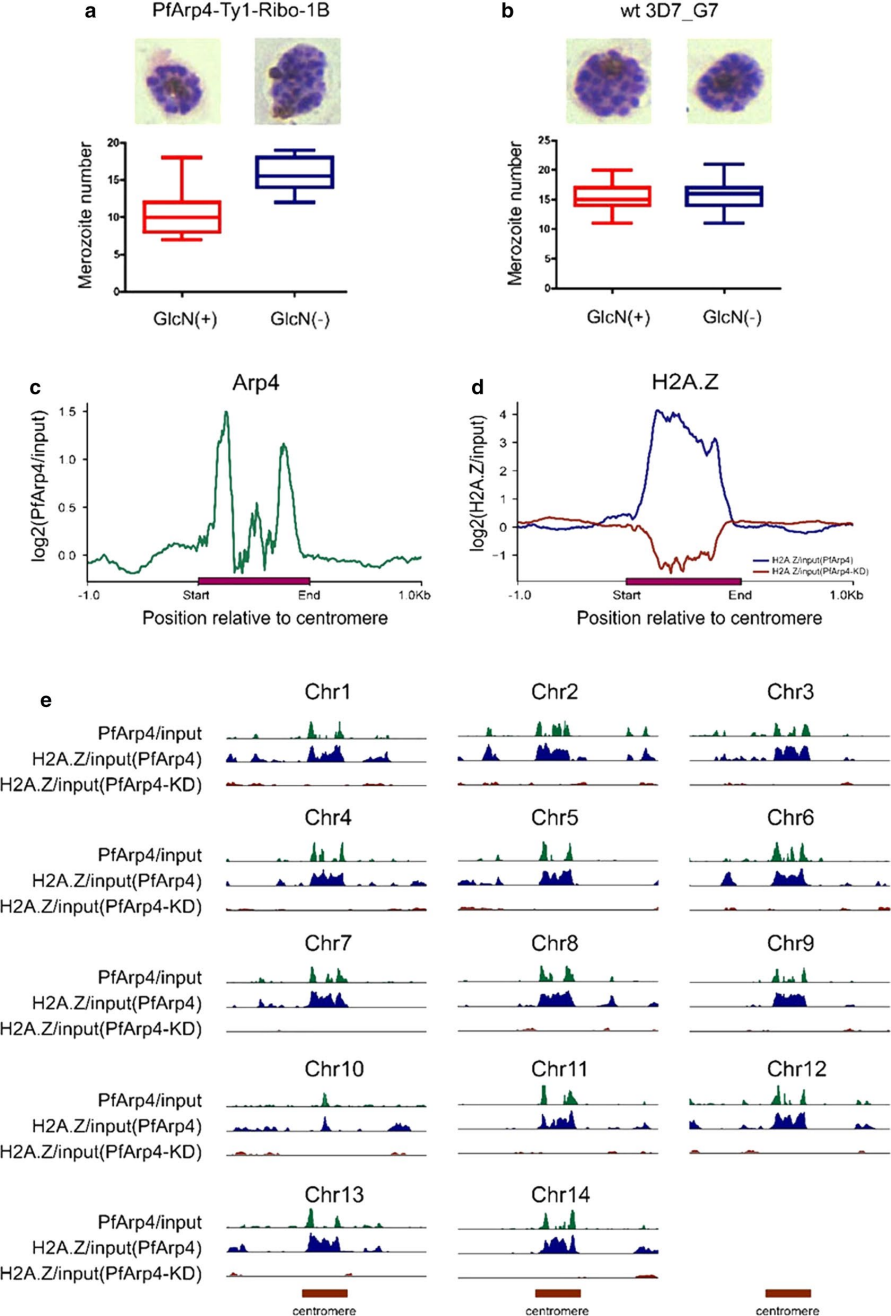
PfArp4 is involved in the centromere biology. **a** Average number of merozoites per schizont in PfArp4-Ty1-Ribo lines cultured with *versus* without GlcN. (Mann-Whitney U-test: *U*_(50)_ = 106.5, *Z* = − 7.93, *P* < 0.0001). Upper: representative Giemsa-stained parasite at late schizont stage. **b** Average number of merozoites per schizont in Pf3D7-G7 lines cultured with *versus* without GlcN. (Mann-Whitney U-test: *U*_(50)_ = 1120, *Z* = − 0.906, *P* = 0.365). Upper: representative Giemsa-stained parasite at late schizont stage. **c** Average profile of PfArp4 enrichment at ring stage (log2[PfArp4 ChIP-normalized read coverage/input-normalized read coverage])relative to centromere for all chromosomes. **d** Average profile of H2A.Z enrichment at ring stage (log2[H2A.Z ChIP-normalized read coverage/input-normalized read coverage])relative to centromere for all genes with (PfArp4 KD H2A.Z/input) or without (PfArp4 H2A.Z/input) GlcN for PfArp4 knockdown. **e** Track view showing the signals atindividual centromeres of PfArp4 (log2[PfArp4/input]) and H2A.Z with (log2[PfArp4 KD H2A.Z/input]) or without (log2[PfArp4 H2A.Z/input]) GlcN for PfArp4 knockdown

Next, we evaluated the knockdown effect of the two transgenic parasite lines by adding of 2.5 mM GlcN. First, we carried out a growth curve assay of the two parasite lines in the blood-stage cycle. The growth of more than 50% of the PfArp4-Ty1-Ribo parasites was arrested when the PfArp4 protein was downregulated, whereas no apparent difference was observed in the PfArp6-Ty1-Ribo line with or without GlcN addition (Fig. 1c, d). The WT 3D7_G7 line was found to have a growth period of ~ 48 h, but the PfArp4 knockdown line had a growth period of ~ 54 h. According to merozoite count tests (Fig. 2a, b), the PfArp4-Ty1-Ribo line had a reduced number of S-stage parasites. Consistent with the results of the growth curve analysis, western blot (WB) analysis showed that the expression level of the PfArp4 protein was significantly reduced in the trophozoite stage parasites when GlcN was present in the culture (Fig. 1e). An abnormal morphology was also observed in the trophozoite-stage parasites of the PfArp4-Ty1-Ribo line treated with GlcN after one cell cycle (Fig. 1f). However, no significant knockdown effect was detected in the parasites of the PfArp6-Ty1-Ribo line, according to the growth curve assay and WB analysis (Additional file 5: Figure S4c, d), indicating that these parasites likely escaped the ribozyme knockdown system.

### PfArp4 and PfArp6 partially colocalized in *P. falciparum*

In *P. falciparum*, PfArp4 and PfArp6 were the two predicted orthologues of nuclear eukaryotic ARPs, but their spatial distribution in the nucleus were unknown. To address this issue, we generated a dually labelled transfectant, PfArp4-Ty1::PfArp6-HA, by using the wild-type 3D7 parasites as the parent line for the transfection (Fig. 1g). Western blot analysis confirmed that the two proteins had been fused with different tags (Additional file 5: Figure S4e). Then, we performed co-IFA and co-immunoprecipitation (co-IP) assays to validate the direct interaction between PfArp4 and PfArp6. As shown in Fig. 1h, this indicates that the two ARPs may be localized to the nucleus although more careful analysis is needed to conclude this throughout blood-stage development. Moreover, the co-IP analysis showed that PfArp6 was pulled down by PfArp4 directly, suggesting that they may be subunits of the same nuclear Arp complex (Fig. 1i).

### PfArp4 knockdown altered the global transcriptome

**Fig. 3:**
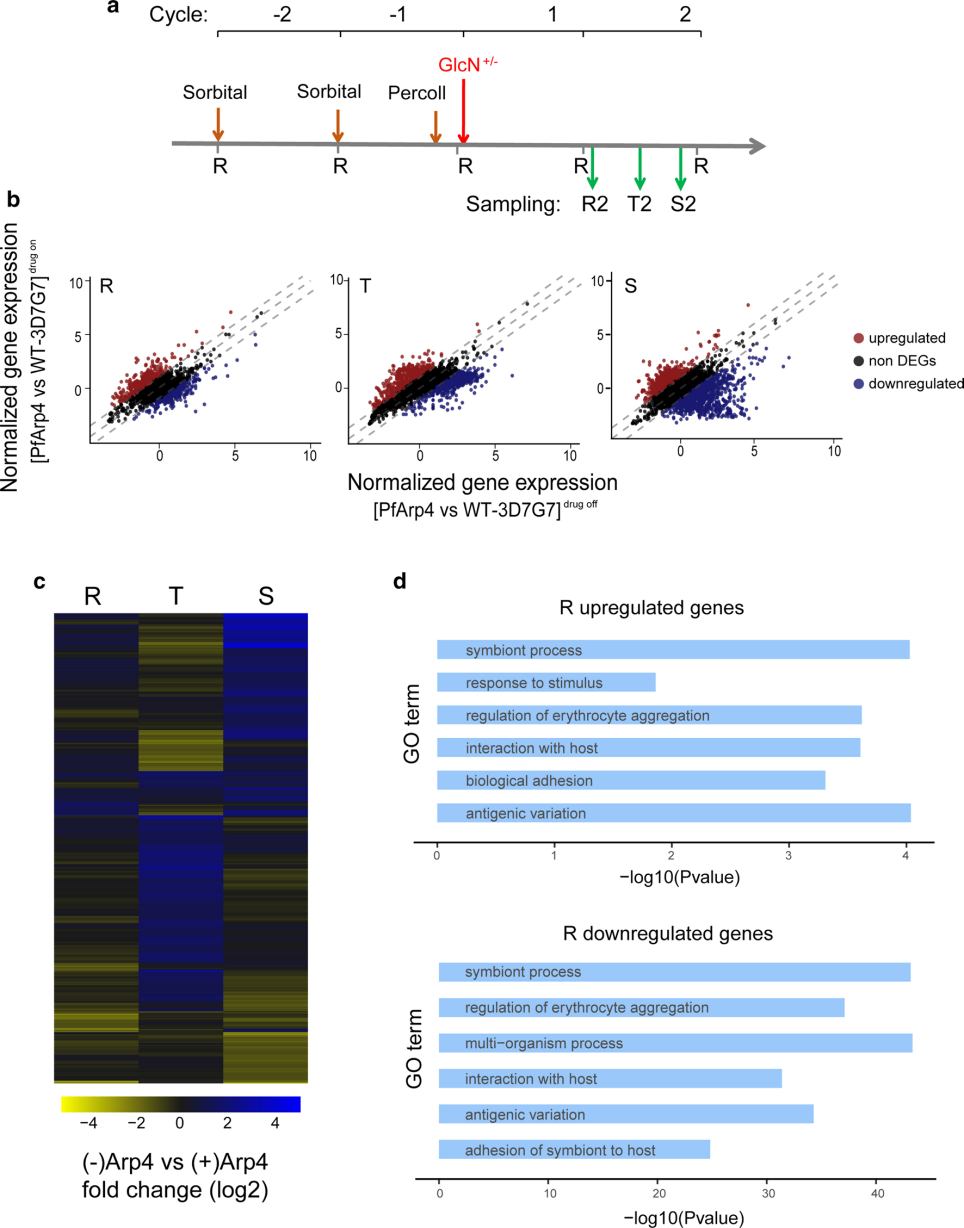
PfArp4 knockdown altered the global transcriptome. **a** The experimental design of parasite treatment and sampling for RNA-seq analysis. The highly synchronized parasites of PfArp4-Ty1-Ribo clone (7A) were harvested in the second cycle after exposure to GlcN for one cycle. Sampling: ring (R2, 8–12 h), trophozoite (T2, 28–32 h), schizont (S2, 40–44 h). **b** Scatterplots showing the comparative transcriptomes of PfArp4-Ty1-Ribo line with *versus* without GlcN at R, T, and S stage, respectively. Fold change (FD) ≥ 2 are used to differentiate the upregulated or downregulated genes. **c** Heatmap showing the fold change (log2) of gene expression upon PfArp4 knockdown across the R, T and S stage, respectively. **d** Enriched Gene Ontology (biological processes) terms for the genes differentially expressed in PfArp4 knockdown line at ring stage

To investigate the effect of PfArp4 protein knockdown on gene expression, we performed comparative transcriptome analyses of the PfArp4-Ty1-Ribo clone (7A) with and without GlcN treatment. The parasites were pre-synchronized twice by sorbitol treatment followed by Percoll enrichment of the late-stage parasites. In the third cycle, the culture was divided into two groups for drug treatment: treated (GlcN^+^) and untreated (GlcN^-^). The parasites at the ring, trophozoite and schizont stages that retained normal morphology in the cell cycle after drug treatment were harvested for RNA-seq (Fig. 3a, Additional file 6: Figure S5). Wild-type 3D7-G7 clones [27] were used as controls for transcriptomic normalization of GlcN treatment.

The comparative transcriptome analysis showed that, by inducible knockdown of the PfArp4 protein, a total of 106, 708 and 638 genes were upregulated twofold or more in the ring, trophozoite and schizont parasites, respectively. In addition, a total of 209, 231 and 296 genes were downregulated more than twofold at the three blood stages, respectively (Fig. 3b, Additional file 7: Table S2). Scatter plots (Fig. 3b) show the comparative transcriptomes of the PfArp4-Ty1-Ribo line with and without GlcN at the R, T and S stages. GO term analysis of the differentially expressed genes showed that a variety of genes corresponding to various biological processes were dysregulated by PfArp4 knockdown (Fig. 3c, d). This result suggests that PfArp4 is involved in the extensive regulation of the dynamic asexual-stage transcriptome of *falciparum* parasites.

### PfArp4 regulated euchromatic gene expression by mediating H2A.Z deposition and H3K9ac modification at promoters

**Fig. 4:**
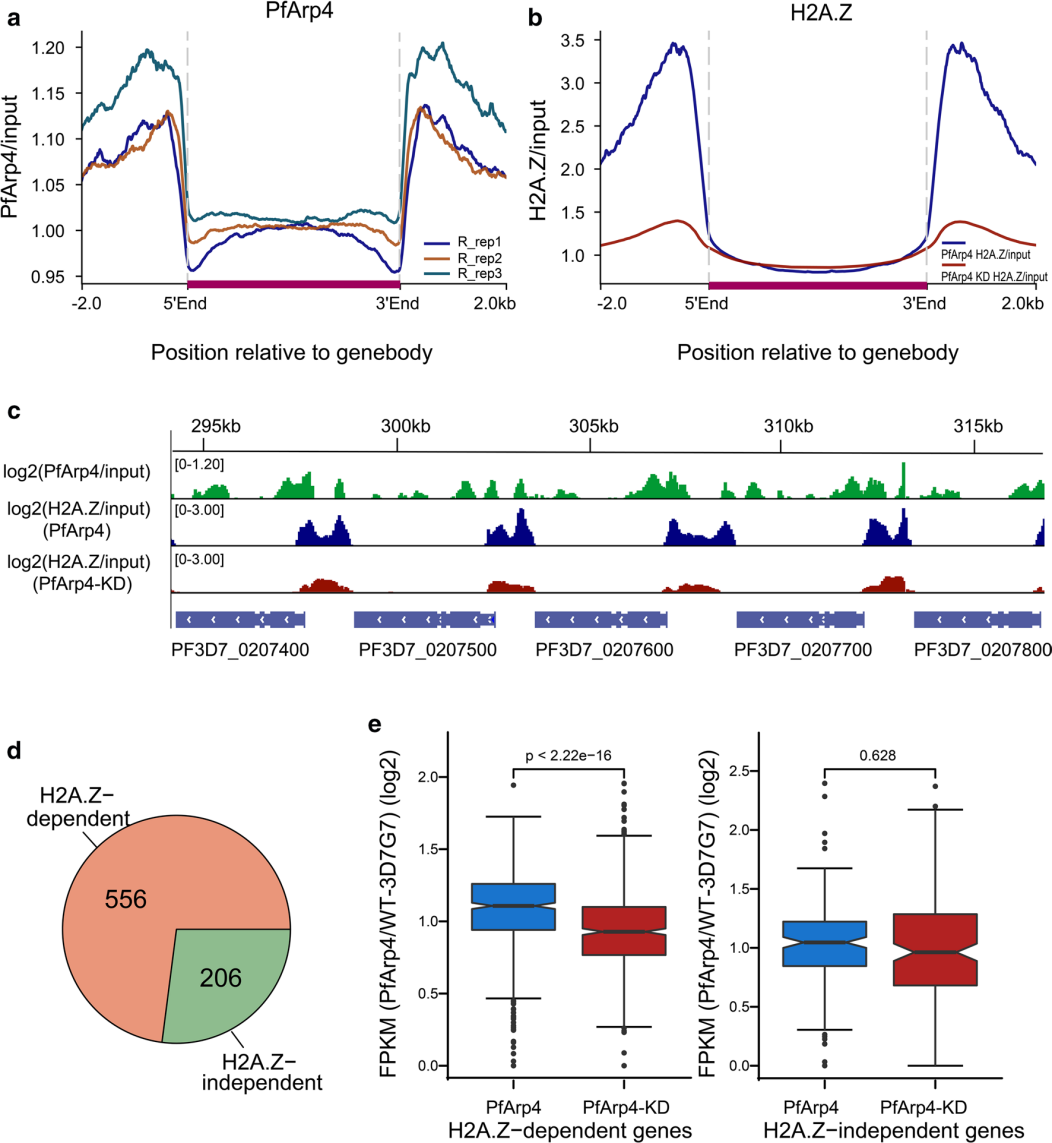
PfArp4 regulated H2A.Z deposition in eukaryotic intergenic regions in the genome. **a** Average profile of PfArp4 enrichment at ring stagerelative to genebody for all genes by three ChIP-seq replications.The data are shown as read coverage of PfArp4 ChIP value normalized with input. **b** Average profile of H2A.Z enrichment at ring stage (H2A.Z ChIP-normalized read coverage/input-normalized read coverage) relative to genebody for all genes of upon PfArp4 knockdown. **c** Normalized read coverage plot of reads mapped to a region of chromosome 2 highlighting H2A.Z is colocalized with PfArp4 and reduced upon PfArp4 knockdown. **d** Pie chart showing the composition of PfArp4-enriched genes with regard to the existence of H2A.Z in genespromoters. **e** Boxplot showing the H2A.Z-dependent genes are significantly downregulated after PfArp4 knockdown (left) while the H2A.Z-independent genes are not (right). Wilcoxon signed-rank test, *Z* = − 8.381; *P*-value < 2.2e-16 (left); Wilcoxon signed-rank test, *Z* = − 0.484, *P* = 0.628 (right)

To determine which group of genes was directly regulated by PfArp4, we carried out ChIP-seq analysis of the synchronized ring-stage (10–20 hpi) parasites from the PfArp4-Ty1-Ribo line through the use of an anti-Ty1 antibody. The genome-wide distribution of PfArp4 ChIP signals showed that the majority of PfArp4 proteins were enriched at the intergenic regions, which generally corresponded to the promoter regions of 762 target genes (Fig. 4a, Additional file 8: Figure S6, Additional file 9: Table S3). A previous study [42] showed that the histone variant H2A.Z demarcated the euchromatic intergenic regions marked dynamically by H3K9ac and H3K4me3, and a significant correlation between mRNA abundance and H2A.Z deposition or H3K9ac level was observed during intraerythrocytic development of *P. falciparum*. Consistent with this finding, we found that the majority of PfArp4 proteins colocalized with the histone variant H2A.Z in the genome of *P. falciparum*, and the conditional knockdown of PfArp4 triggered the dramatic removal of H2A.Z from the upstream regions of the eukaryotic genes (Fig. 4b, c).

To evaluate the direct regulatory function of PfArp4 on H2A.Z-associated euchromatic genes, we classified the PfArp4-enriched genes in the ring-stage parasites into two subgroups, i.e. H2A.Z-dependent and H2A.Z-independent (556 *versus* 206) genes on the basis of the ChIP signal distribution (Fig. 4d). Statistical analysis showed that H2A.Z-dependent genes were significantly downregulated upon PfArp4 knockdown, whereas no difference was observed for the H2A.Z-independent genes (Fig. 4e). This result suggests that PfArp4 positively regulates the transcription of euchromatic genes by mediating H2A.Z deposition at promoter regions.

**Fig. 5:**
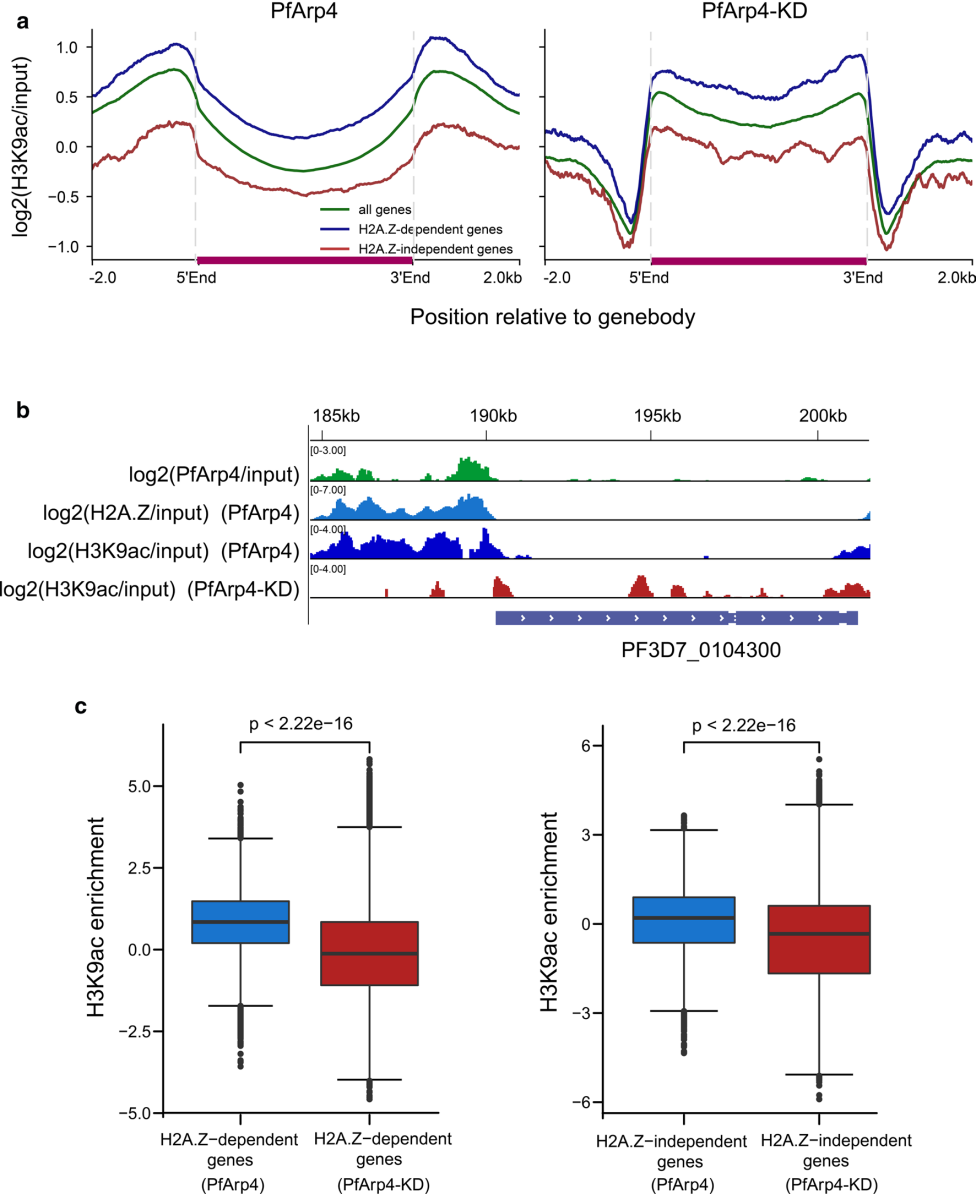
PfArp4 knockdown disrupted the H3K9ac distribution at euchromatic gene loci. **a** Average profile of H3K9ac enrichment (log2[H3K9ac ChIP-normalized read coverage/input-normalized read coverage]) of PfArp4-Ty1-Ribo line with (right) *versus* without (left) GlcN at ring stage for H2A.Z-dependent or H2A.Z-independent genes, respectively. **b** Track view showing the H3K9ac signals in one representative H2A.Z-dependent gene PF3D7_0104300. **c** Boxplot showing the H3K9ac enrichment (log2[H3K9ac ChIP-normalized read coverage/input-normalized read coverage]) of H2A.Z-dependent genes (left) and H2A.Z-independent genes (right) are both significantly decreased with PfArp4 knockdown. Wilcoxon signed-rank test, *Z* = − 168.311; *P*-value < 2.2e-16; Wilcoxon signed-rank test, Z = − 63.162 (left); *P*-value < 2.2e-16 (right)

In addition, we used a ChIP-seq assay to analyse the alteration of H3K9ac modification upon PfArp4 knockdown. As shown in Fig. 5a (left), H2A.Z-dependent genes and H2A.Z-independent genes were marked by H3K9ac with regard to the ChIP signal distribution in both the 5′ UTR and 3′ UTR. As expected, the H3K9ac levels at the untranslated regions of either of the two groups were reduced in the ring-stage PfArp4-knockdown parasites. Strikingly, for the gene-coding regions, the H3K9ac mark was enhanced compared to that in the parasites with normal PfArp4 levels (Fig. 5a, b). This result suggests the cooperation of PfArp4 and H2A.Z at the untranslated regions may demarcate the euchromatic intergenic regions in the genome as chromatin boundaries, which was disrupted by the removal of PfArp4 and H2A.Z and the subsequent chromatin remodelling they induce. Statistical analysis showed that both classes of H2A.Z-dependent and H2A.Z-independent genes were downregulated upon PfArp4 knockdown, but the latter exhibited fewer changes (Fig. 5c). This result confirms the critical role of the PfArp4 and H2A.Z partnership in the transcriptional regulation of euchromatic genes.

### PfArp4 is involved in the centromere function in *P. falciparum*

In other eukaryotes, Arp4 functions in centromere biology, including kinetochore-spindle attachment, kinetochore assembly, chromosome segregation, and gene silencing at the centromere. To determine the role of the orthologue in malaria parasites, we measured the ChIP signal of the PfArp4 protein at the centromeric regions in unicellular ring-stage parasites. Intriguingly, in contrast to the profile of Arp4 localization at the centromere in the budding yeast *Saccharomyces cerevisiae*, PfArp4 was preferentially localized to both sides of all 14 centromeres (Fig. 2c). For *P. falciparum*, a previous study had shown that the centromeric regions are marked by H2A.Z instead of H3K9me3, which is as the mark typically observed in eukaryotes. Here, as in other chromosomal regions, the H2A.Z deposition at the centromere was almost abolished when the PfArp4 protein was downregulated (Fig. 2d, e). This finding suggests that the PfArp4 protein likely contributes to the formation or functions as a boundary marker for the unique centromeric chromatin structure in malaria parasites.

Finally, the deposition of PfArp4 at the flanking sites of centromeres may be involved in chromosome segregation and replication, thereby regulating the schizogony process of intraerythrocytic parasites, i.e. the transition from trophozoite to schizont. Thus, we counted the average number of merozoites per individual late-stage schizont of the PfArp4-Ty1-Ribo line with and without GlcN. As expected, the merozoite number was significantly reduced in the PfArp4-knockdown parasites, whereas no difference was observed in the WT parasites (Fig. 2a, b). This finding may partially explain the growth arrest of PfArp4-knockdown parasites (Fig. 1f). Together, these data demonstrated that PfArp4 has roles in centromere biology, including involvement in local chromatin structure and parasite proliferation.

## Discussion

The actin family consists of canonical actin(s) and actin-related proteins (ARPs) that are highly conserved in the composition and sequences in various eukaryotes. To date, multiple members that are localized predominantly in the nucleus were identified after they were initially found in various ARPs in the cytoplasmic compartment of cells. While these cytoplasmic ARPs are involved in structural functions, nuclear ARPs execute additional functions, such as gene regulation, by participating in ATP-dependent chromatin remodelling and histone acetyltransferase complexes [43]. In addition, recent studies have shown that nuclear ARPs, e.g. yeast Arp6, regulate high-order nuclear organization [44, 45]. Therefore, nuclear ARPs are now recognized as newly discovered regulators of dynamic gene expression and nuclear architecture during various physiological processes. In this study, using results from previous bioinformatics analyses [26], we characterized the two putative nuclear ARPs in *P. falciparum* for the first time, i.e. PfArp4 and PfArp6. Biochemical analysis demonstrated the colocalization of these two nuclear PfARPs. Because the *PfArp6* gene was not downregulated by the ribozyme system, we were unable to study the role of PfArp6 in gene regulation and its related mechanisms. Usually, using the glucosamine-induced glmS-ribozyme system to control the expression of *P. falciparum* target genes bearing a glmS ribozyme in the 3′ untranslated region is efficient. For PfArp6, we found that this mechanism did not work well in our experiment. It seems that the efficiency of this system is dependent on different genes. Determining whether this phenomenon affects other *P. falciparum* genes is worthy of further study. Eukaryotic Arp6 has been shown to regulate nuclear organization, which may confer additional regulatory functions that are not observed for PfArp4.

Importantly, our data strongly support PfArp4 as a critical regulator positively correlated with euchromatic gene expression because it mediates H2A.Z deposition and H3K9ac modification in *P. falciparum* [42]. In other eukaryotes, the histone variant H2A.Z is involved in transcriptional regulation of a wide range of genes by replacing the canonical histone H2A at promoter regions [46]. Study on the mechanisms in either the turnover or stabilization of H2A.Z-containing nucleosomes will contribute to the understanding of this complex regulatory network of gene expression in eukaryotes. In human malaria parasites, high-throughput ChIP-seq analysis revealed that the histone variant H2A.Z demarcates the euchromatic intergenic regions mostly associated with the upstream promoter region of the target genes. Integrated transcriptome analysis and ChIP-seq data showed that H2A.Z coupled with H3K9ac marked the transcriptional activity of the promoters in the genes for which they are enriched [42]. Thus, together with the predominant heterochromatic marker H3K9me3 or HP1 [47], they likely mark the dynamic epigenome in malaria parasites. Moreover, H2A.Z and H2B. Z double-variant nucleosomes have been shown to define intergenic regions and are dynamically localized at the promoters of the primary virulence gene (*var*) promoters in *P. falciparum* [48, 49]. Here, our experimental evidence showed that PfArp4 is associated with H2A.Z deposition at eukaryotic promoters, which provides mechanistic insight into the stabilization and turnover of H2A.Z-containing nucleosomes in eukaryotic cells.

In addition, we found that PfArp4 also occupied the two flanking sites of centromeres, likely as a boundary mark for this special chromatin structure, which is enriched by H2A.Z instead of heterochromatic markers, in contrast to the marks in other eukaryotes [42]. The knockdown of PfArp4 triggered the depletion of H2A.Z from all centromeric regions; however, with the data available, it was unclear whether local centromeric chromatin remodelling influenced the transcriptome of these parasites. In yeast, Arp6 is able to mediate H2A.Z/Htz1-dependent and H2A.Z/Htz1-independent chromatin binding to nuclear pores to modulate gene expression [45]. However, the centromeric localization of eukaryotic Arp4 is most likely associated with kinetochore assembly and chromosome segregation, thereby controlling cell development and proliferation [50]. Growth arrest accompanied by reduced schizogony efficiency upon *PfArp4* gene knockdown further supports the function of the PfArp4 protein during the nuclear division of malaria parasites.

## Conclusions

Here, we discovered that PfArp4 correlated positively with the dynamic expression of eukaryotic genes. The PfArp4 protein colocalized with the histone variant H2A.Z and euchromatic marker H3K9ac in the intergenic regions. Inducible downregulation of PfArp4 resulted in the depletion of H2A.Z and lower H3K9ac levels at the upstream regions of eukaryotic genes, thereby repressing the transcriptional abundance of H2A.Z-dependent genes. Intriguingly, PfArp4 was localized at the flanking sites of all centromeres, likely shaping the H2A.Z-enriched centromeric chromatin structure as a boundary. PfArp4 depletion triggered the loss of H2A.Z at all centromere regions and arrested blood-stage development, probably by interfering with nuclear division in the schizogony process. Taken together, our findings suggest that PfArp4 regulates the cell cycle by controlling H2A.Z deposition and affecting centromere function, which contributes to the understanding the complex epigenetic regulation of gene expression and development of malaria parasites.

## Supplementary information

**Additional file 1: Table S1.** Accession numbers of Arp4 and Arp6.

**Additional file 2: Figure S1.** Phylogenetic trees of PfArp4 and PfArp6 orthologs in eukaryotes. The unique clade of the genus *Plasmodium* is indicated by a red dash box.

**Additional file 3: Figure S2.** A multiple sequence alignment of Arp4.

**Additional file 4: Figure S3.** A multiple sequence alignment of Arp6.

**Additional file 5: Figure S4.** Knockdown approach of PfArp6 **a** Schematic representation of generation of PfArp6-Ty1-Ribo transgenic parasites with the same strategy shown in Fig. 1a. **b** PCR analysis of PfArp6-Ty1-Ribo lines. The sequences of primer pairs were listed in Additional file 10: Table S4. **c** Growth curve assay of PfArp6-Ty1-Ribo lines with versus without GlcN drug in the culture. The parasitaemia were measured by microscopy (*n* = 3, bars are SD). **d** Western blot of ring (R2, 8–12 h), trophozoite (T2, 28–32 h) and schizont (S2, 40–44 h) stage parasites of PfArp6-Ty1-Ribo line with or without GlcN in culture for one cycle. **e** Western blot analysis of PfArp4-Ty1::PfArp6-HA lines with antibody against Ty1 and HA epitope, respectively. The protein extracts of PfArp6-HA-Ty1 and PfArp4-Ty1-Ribo were used as controls. The aldolase signal was used as the internal control.

**Additional file 6: Figure S5.** Reproducibility of RNA-seq data of PfArp4-Ty1-Ribo line throughout the blood-stage developmental cycle **a** Correlation of FPKM at R, T, S stages of parasite with wild-type PfArp4 between two biological replicates. Pearson correlation between biological replicates are displayed at top left corner. Correlation: *P*-value < 2.2e-16, *r*_(5712)_ = 0.953; *P*-value < 2.2e-16, *r*_(5712)_ = 0.979; *P*-value < 2.2e-16, *r*_(5712)_ = 0.980. **b** Correlation of FPKM at R, T, S stages upon PfArp4 knockdown between two biological replicates. Pearson correlation between biological replicates is displayed at top left corner. Correlation: *P*-value < 2.2e-16, *r*_(5712)_ = 0.912; *P*-value < 2.2e-16, *r*_(5712)_ = 0.959; *P*-value < 2.2e-16, *r*_(5712)_ = 0.895.

**Additional file 7: Table S2.** FPKM values of RNA-seq analysis for PfArp4-Ty1-Ribo line with or without GlcN in culture for knockdown.

**Additional file 8: Figure S6.** Reproducibility of PfArp4 ChIP-seq at ring stage. Heatmap showing the correlation of genic ChIP signals between three biological replicates. Pearson correlation is displayed in the heatmap.

**Additional file 9: Table S3.** A list of genes enriched for PfArp4 in the promoter region.

**Additional file 10: Table S4.** Sequences of primer pairs used to construct and verify PfArp4 or PfArp6 transgenic parasite lines.

## Data Availability

Data supporting the conclusions of this article are included within the article and its additional files. The high-throughput sequencing data of this study have been deposited in Gene Expression Omnibus (GEO) database under accession number GSE143902.

## Abbreviations

ARP: Actin-related protein
Var: Variant antigen
HA: Haemagglutinin
GlcN: Glucosamine
IGV: Integrative genomics viewer
IP: Immunoprecipitation
IFA: Immunofluorescence assays
BSD: Blasticidin-S deaminase
