# Supplementary figures and images for: Actin-related protein Arp4 regulates euchromatic gene expression and development through H2A.Z deposition in blood-stage Plasmodium falciparum
Source: Parasit Vectors. 2020 Jun 17;13:314. doi: 10.1186/s13071-020-04139-6 (PMC7301494; doi:10.1186/s13071-020-04139-6)

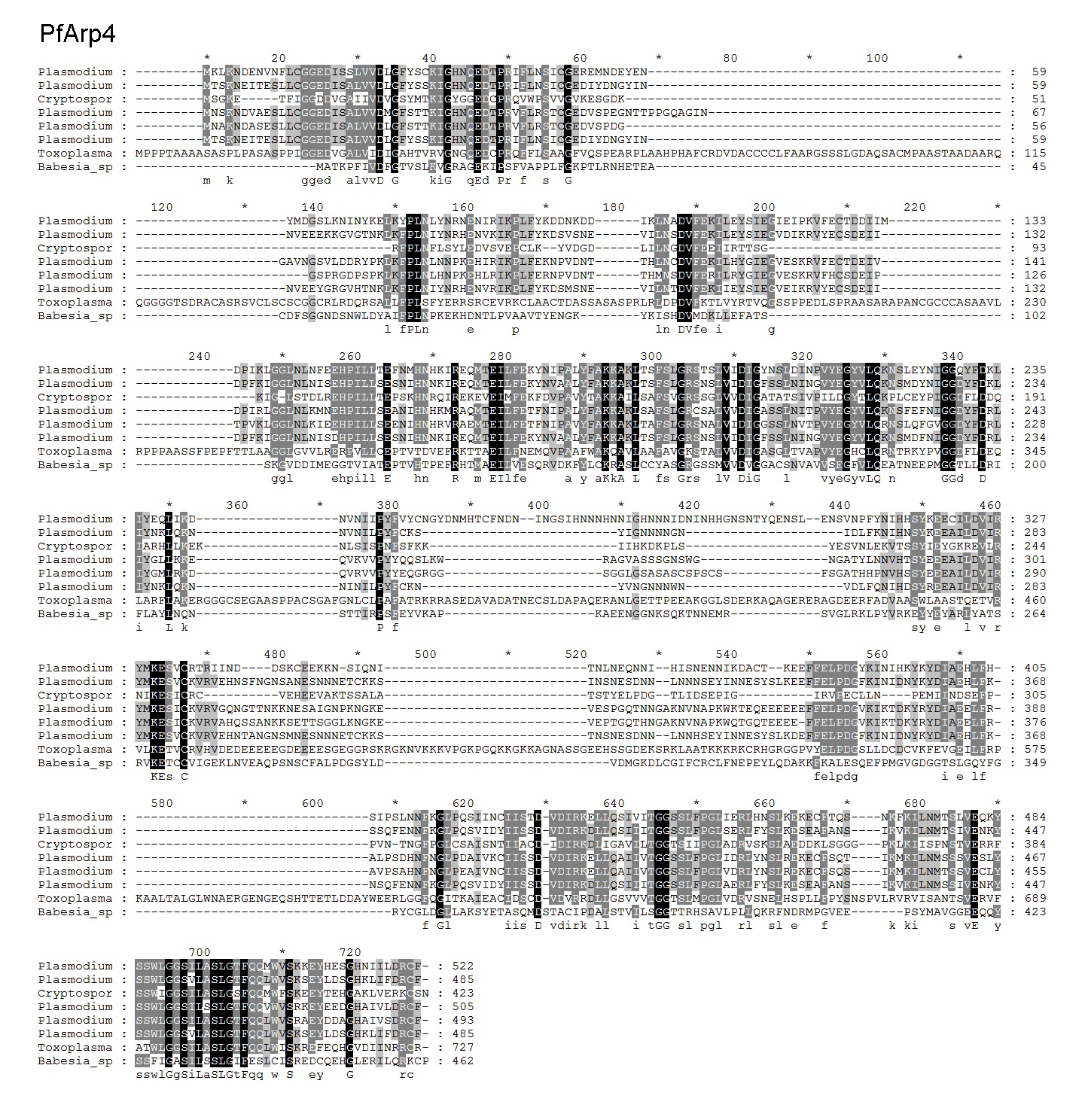

Supplement: Supplementary file 3 — Additional file 3: Figure S2. A multiple sequence alignment of Arp4. [file 13071_2020_4139_MOESM3_ESM.tif]

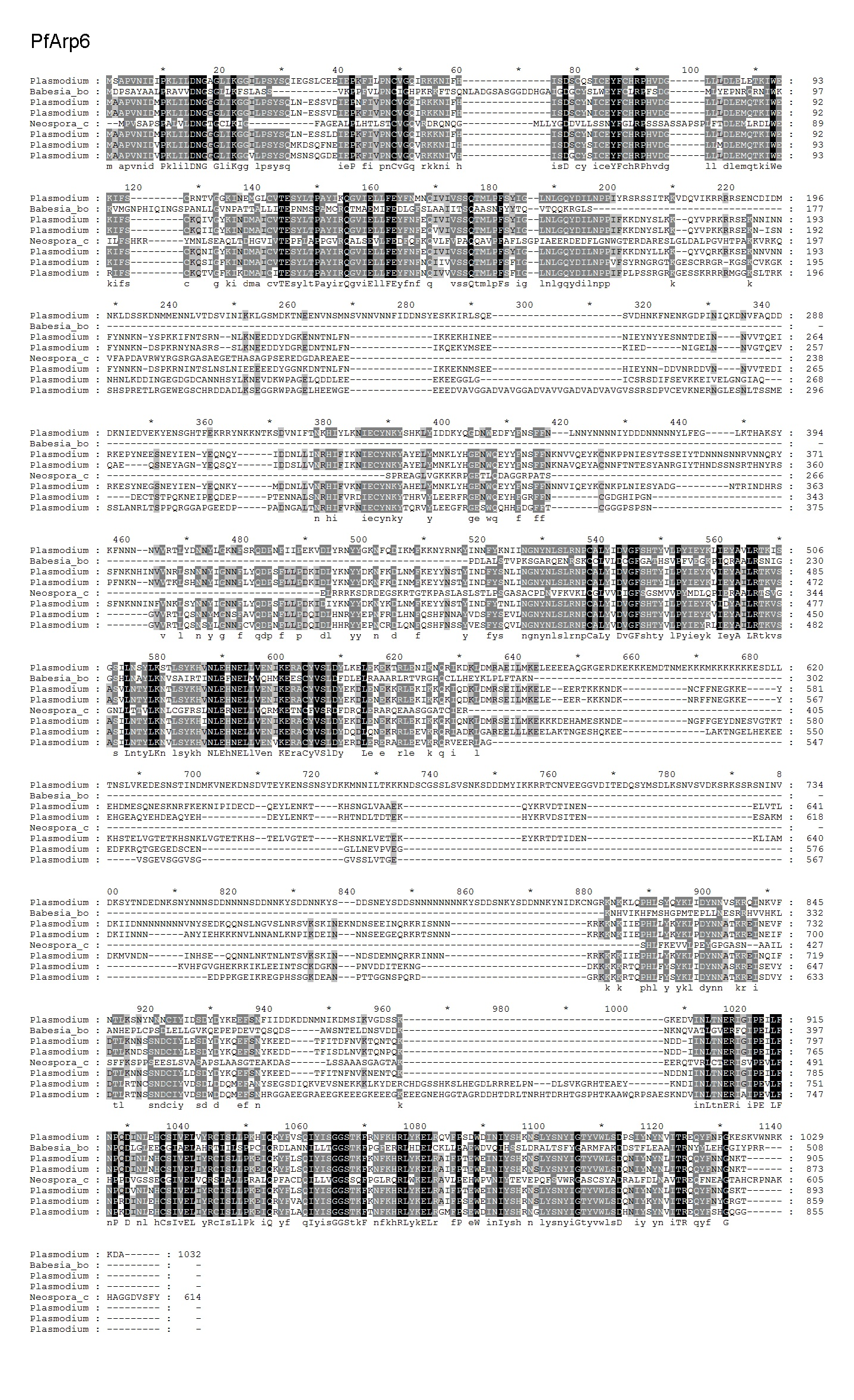

Supplement: Supplementary file 4 — Additional file 4: Figure S3. A multiple sequence alignment of Arp6. [file 13071_2020_4139_MOESM4_ESM.tif]
